# Mitochondrial and plastid genome analysis of the heteromorphic red alga *Mastocarpus papillatus* (C. Agardh) Kützing (Phyllophoraceae, Rhodophyta) reveals two characteristic florideophyte organellar genomes

**DOI:** 10.1080/23802359.2016.1219636

**Published:** 2016-09-03

**Authors:** Marina Nasri Sissini, Teresa M. Navarrete-Fernández, Erin M.C. Murray, Jillian M. Freese, Anais S. Gentilhomme, Soren R. Huber, Thomas F. Mumford, Jeffery R. Hughey

**Affiliations:** a Programa de Pós-graduação em Ecologia, Universidade Federal de Santa Catarina, Campus Trindade, Florianopolis, Santa Catarina, Brazil;; b Estación Costera de Investigaciones Marinas, Pontificia Universidad Católica de Chile, Santiago, Chile;; c Friday Harbor Laboratories, University of Washington, Friday Harbor, Washington, USA;; d Department of Biological Sciences, University of Rhode Island, Kingston, RI, USA;; e Division of Mathematics, Science, and Engineering, Hartnell College, Salinas, CA, USA

**Keywords:** Gigartinales, Mastocarpus, mitogenome, Phyllophoraceae, plastid genome

## Abstract

Analysis of the marine red algal species *Mastocarpus papillatus* (C. Agardh) Kützing using paired-end 36 bp Illumina sequences resulted in the assembly of its complete mitochondrial and plastid genomes. The mitogenome is 25,906 bp in length and contains 50 genes, and the plastid genome is 184,382 bp with 234 genes. The mitochondrial and plastid genomes show high gene synteny with published Florideophyceae.

The marine red algal family Phyllophoraceae Nägeli has a worldwide distribution and consists of 14 genera and approximately 123 species (Schneider & Wynne 2007; Guiry & Guiry 2016). One of these genera, *Mastocarpus* Kützing, is currently circumscribed to accommodate species with heteromorphic life histories in which upright gametophytes (with carposporophytes) alternate with crustose tetrasporophytes previously known as *Petrocelis* (Guiry et al. 1984). The life history has also been shown to be apomictic (Polanshek & West 1977). *Mastocarpus* currently contains 15 well-defined species, however, no mitochondrial or plastid genomes have been reported for the family. Here, we describe the organellar genomes of *M. papillatus*, a well studied entity distributed from northern Vancouver Island, British Columbia to Cambria, California (Polanshek & West 1977; Lindstrom et al. 2011).

DNA was extracted from *M. papillatus* (Specimen Voucher- UC2050562) collected from the high intertidal at Baker Beach, San Francisco, California (37°47′54.1″N, -122°28′53.9″W), the species type locality, following the protocol of Lindstrom et al. (2011). The library construction and sequencing was performed by the High-Throughput Genomics Center (Seattle, Washington) yielding 17,782,094 filtered reads. The data were assembled using default *de novo* settings in CLC Genomics Workbench 9.0 (^®^2016 CLC bio, a QIAGEN Company, Waltham, MA) and annotated following Hughey et al. (2014). Alignment of the *M. papillatus* mitogenome to other Florideophyceae was accomplished with MAFFT (Katoh & Standley 2013). The maximum likelihood analysis was performed using complete mitogenome sequences in RaxML (Stamatakis 2014) with Galaxy (Giardine et al. 2005; Blankenberg et al. 2010; Goecks et al. 2010) set to 1000 fast bootstraps with the GRT + gamma model. The tree was visualized with TreeDyn 198.3 at Phylogeny.fr (Dereeper et al. 2008).

The mitogenome of *M. papillatus* (GenBank KX525587) is 25,906 bp in length, AT skewed (65.0%), and contains 50 genes including 22 tRNA (trnG, trnL, trnM, trnR occur in duplicate, trnS occurs in triplicate), five ribosomal proteins (*rpl 16*, *rpl 20*, *rps 3*, *rps 11*, *rps 12*), 2 rRNA (*1 rnl*, *1 rns*), *tatA*, and 19 other genes involved in electron transport and oxidative phosphorylation. The mitogenome is highly conserved in organization compared to other Florideophyceae (Yang et al. 2015). Phylogenetic analysis of *M. papillatus* resolves it in a strongly supported clade with *Chondrus crispus* Stackhouse (Figure 1).

**Figure 1.**
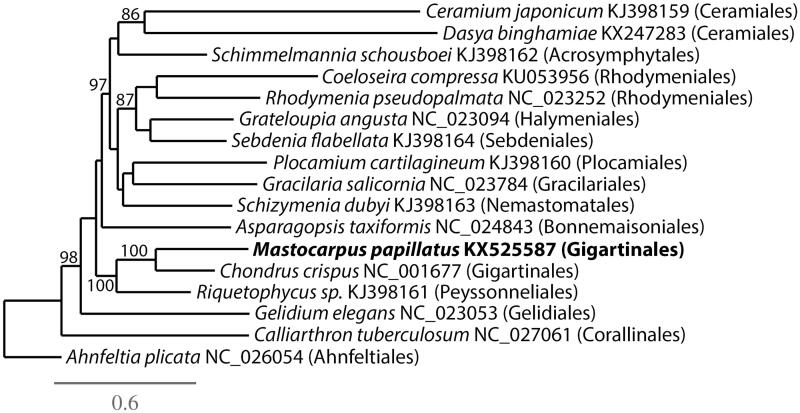
Maximum-likelihood phylogram with *M. papillatus* and other Florideophyceaen mitogenomes. Numbers along branches are RaxML bootstrap supports based on 1000 nreps (<75% support not shown). The legend below represents the scale for nucleotide substitutions.

The complete plastid genome of *M. papillatus* (GenBank KX525588) is 184,382 bp in length and contains 234 genes. The genome is AT rich (70.9%) and contains 47 ribosomal proteins, 36 ycf, 30 photosystem I and II, 29 tRNA, 10 phycobiliprotein, 10 cytochrome b/f complex, 9 ATP synthase, 5 RNA polymerase, 3 orf, 3 rRNA, and 52 other genes. Examination of the *M. papillatus* plastid gene content and organization indicates high-gene synteny to other Rhodymeniophycidae.
